# Guideline implementation and early risk assessment in pulmonary arterial hypertension associated with congenital heart disease: A retrospective cohort study

**DOI:** 10.1111/crj.13076

**Published:** 2019-08-29

**Authors:** Xiaoxian Deng, Bowen Jin, Shanshan Li, Yaping Li, Hongmei Zhou, Yang Wu, Menghuan Yan, Yuanping Hu, Qiu Qiu, Gangcheng Zhang, Xuan Zheng

**Affiliations:** ^1^ Congenital Heart Disease Center, Wuhan Asia Heart Hospital Wuhan University of Science and Technology Wuhan China; ^2^ Imaging Center, Wuhan Asia Heart Hospital Wuhan University of Science and Technology Wuhan China; ^3^ Laboratory of Molecular Cardiology, Wuhan Asia Heart hospital Wuhan University of Science and Technology Wuhan China

**Keywords:** congenital heart disease, guidelines, prognosis, pulmonary arterial hypertension, risk stratification

## Abstract

**Introduction:**

Current guidelines emphasize that accurate risk stratification is important for patients with pulmonary arterial hypertension (PAH), however, few suggestions have been specified for PAH associated with congenital heart disease (PAH‐CHD).

**Objectives:**

The aim of this study was to propose an accurate and simple system based on current guidelines for risk stratification in PAH‐CHD patients during 12‐month follow‐up.

**Methods:**

We reviewed 288 Chinese PAH‐CHD patients between January 2014 and December 2016 in this retrospective cohort study. The low‐risk criteria according to 2015 European Society of Cardiology guidelines and the adverse events (AEs) during follow‐up were collected. The association between low‐risk criteria and AEs was assessed with Cox regression, and a simplified risk stratification system was proposed.

**Results:**

There were 105 PAH‐CHD patients included in the final analysis. Twenty‐nine patients had AEs defined as death, initiation of new or combined medication treatment, or re‐hospitalisation because of the PAH worsening. Among the low‐risk criteria, WHO/NYHA functional class, 6‐minute walking distance (6MWD), NT‐proBNP and SvO_2_ were significantly different between AE and AE‐free groups. However, 6MWD (HR = 0.08, 95% CI: 0.03‐0.19, *P *< 0.001) and NT‐proBNP (HR = 0.35, 95% CI: 0.16‐0.78, *P* = 0.01) were the only independent predictors of AEs in multivariable model. When taking them into a simplified system for risk stratification, the number of low‐risk criteria at diagnosis discriminated the risk of AEs (*P *< 0.001).

**Conclusions:**

Among the low‐risk criteria proposed by current guidelines, 6MWD and NT‐proBNP predicted AEs independently for PAH‐CHD patients. Simplified risk stratification system by taking these two parameters numerically provides accurate prognostic information in PAH‐CHD patients.

## 1 INTRODUCTION

Pulmonary arterial hypertension (PAH) is characterised by progressively increased pulmonary arterial pressure, which results from several different diseases, such as congenital heart diseases, connective tissue disease, chronic thrombotic pulmonary disease and others. Different from the etiology in Western countries, PAH associated with congenital heart disease (PAH‐CHD) accounts for most incident of PAH in Asia.^1^ Similar to idiopathic PAH (iPAH), part of PAH‐CHD patients develops right heart failure because of the irreversible PAH regardless the targeting medicine therapy.^2^

2015 European Society of Cardiology (ESC)/European Respiratory Society (ERS) guidelines for the diagnosis and treatment of pulmonary hypertension recommend a risk assessment including both clinical, biochemical, imaging and hemodynamic parameters.^3^ By separating patients into three groups with low‐, intermediate‐ and high‐risk of poor prognosis, different treatment goals and strategies are meant to achieve. Furthermore, accurate risk stratification for PAH patients at the time of diagnosis will improve their prognosis by starting the personalised therapy as soon as possible.^4^ However, guidelines only offer a general recommendation for PAH patients, there are no specific criteria for subgroups. In addition, most studies focused on iPAH and others, risk assessment for PAH‐CHD still needs further investigations.^5^, ^6^, ^7^, ^8^

In this study, we evaluated the risk assessment criteria from guidelines for Chinese PAH‐CHD patients. The aim was to investigate the predictive value of each parameter from guidelines in PAH‐CHD and to propose a simplified stratification system thereafter for clinical practice. We hypothesised that simplified risk stratification system based on the re‐evaluation of current guidelines would successfully identify PAH‐CHD patients with poor prognosis.

## 2 MATERIAL AND METHODS

### 2.1 Study design

A short‐term retrospective cohort study was conducted in Wuhan Asia heart hospital, Wuhan, China to evaluate the prognostic value of risk assessment based on current guidelines for patients with PAH‐CHD, and a modified system for risk stratification was proposed.

### 2.2 Study population

We reviewed all PAH‐CHD patients who were newly diagnosed in our centre between January 2014 and December 2016. PAH was diagnosed according to right heart catheterisation (RHC), which was defined as a mean pulmonary arterial pressure ≥ 25 mm Hg, pulmonary arterial wedge pressure ≤ 15 mm Hg and pulmonary vascular resistance > 3 Wood units.^3^ Exclusion criteria were (a) patients who were younger than 18; (b) patients whose PAH was reversed after CHD correction; and (c) patients with incomplete clinical data. All patients received proper medical care upon their diagnosis. The study was approved by the Ethics Committee of Wuhan Asia heart hospital with a waiver of informed consent (2014‐P‐001). Personal information of patients was re‐identified before analysis.

### 2.3 Data collection

Clinical data including age, gender, body mass index (BMI), vital signs, WHO/NYHA functional class, 6‐minute walking distance (6MWD) at admission were collected from medical records. Laboratory biomarkers such as white blood cell count (WBC), serum creatinine (Scr), alanine aminostransferase (ALT), aspartate aminotransferase (AST) and N‐terminal prohormone of brain natriuretic peptide (NT‐proBNP) were also collected. These blood tests were analysed in fresh blood and determined by standard quantitative assay techniques in our Department of Pathology and Clinical Laboratory, according to the manufacturer’s protocol. Hemodynamic measurements were obtained during the procedure of RHC at baseline.

Low risk criteria including WHO/NYHA functional class, 6MWD, NT‐proBNP, RAP, CI and SvO_2_% were evaluated as predictors for prognosis, according to 2015 ESC/ERS pulmonary hypertension guideline. Risk assessment was performed thereafter based on the numbers of low risk criteria. Adverse events (AEs) during 12‐month follow‐up were defined as the composite of death, initiation of new or combined medication treatment or re‐hospitalisation because of the PAH worsening. The follow‐up was censored when patients had an AE or were followed up to 12 months.

### 2.4 Statistical analysis

Baseline characteristics are reported as number for categorical data, and mean ± standard deviation or median with interquartile range (IQR) as appropriate for continuous data. Baseline characteristics were compared with student *t* test or Wilcoxon signed‐rank test for continuous variables depending on the normality of their distributions and with the *χ* ^2^ test for categorical variables. A univariate Cox proportional hazard model was used to identify the significant determinants for further multivariate Cox regression analysis. The survival of patients at different risk was illustrated by Kaplan‐Meier survival curve, and compared using log rank *t *test. A two‐side *P *< 0.05 was considered as statistic significant. All statistical analyses were performed with SPSS 23.0 (IBM Corp, Armonk, NY).

## 3 RESULTS AND DISCUSSION

### 3.1 Patient cohort

**Figure 1:**
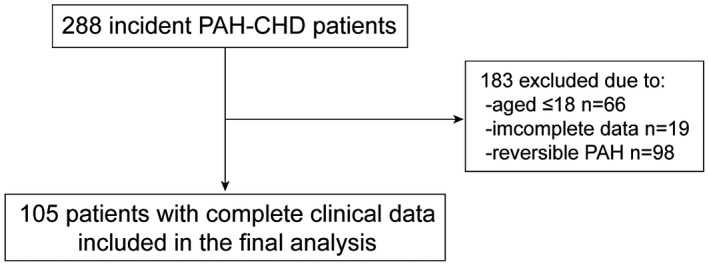
Study flowchart

A total of 288 PAH‐CHD patients were reviewed at the beginning of this study. Figure 1 illustrates the study flow. There were 66 patients aged from 0.5 to 18. Nineteen patients were with incomplete data. Pulmonary artery pressures were decreased to normal after CHD correction in 98 patients. Therefore, 105 PAH‐CHD patients were included in the final analysis. Among the study population, 69 (65.70%) patients were affected by Eisenmenger’s syndrome, 17 (16.20%) were affected by PAH associated with prevalent systemic‐to‐pulmonary shunts, 7 (6.70%) were affected by PAH with small defects and 12 (11.40%) were affected by PAH after defect correction. A total of 29 (27.60%) patients had AEs during follow‐up. Specifically, 11 (37.90%) started new or combined medication therapy, 10 (34.50%) were re‐hospitalised because of the clinical worsening. Eight (27.60%) patients died. The demographic and clinical characteristics of the study population, as well as laboratory and hemodynamic measurements, are presented in Table 1. Gender, age and BMI were similar between AE and AE‐free groups. The incidences of AEs, which mostly came from Eisenmenger’s syndrome (65.50%), were similar between subgroups, however, death were seen less in Eisenmenger’s syndrome (4, 21.10%) when compared with others (*P *< 0.001). WHO/NYHA functional class, 6MWD, NT‐proBNP, mean pulmonary arterial pressure (mPAP), CI and SvO_2_% were significantly different between AE and AE‐free groups.

**Table 1:** Demographics and baseline characteristics of patients with PAH‐CHD

	AE‐free	AE	*P* value
Subjects	76	29	−
Female (*n*)	25	13	0.255
Age (years)	38 ± 13	37 ± 12	0.575
BMI (kg/m^2^)	20.5 ± 3.9	20.6 ± 3.8	0.926
PAH diagnosis (*n*)			
Eisenmenger’s syndrome	50	19	0.423
PAH associated with prevalent systemic‐to‐pulmonary shunts	12	5	
PAH with small/coincidental defects	5	2	
PAH after defect correction	9	3	
WHO/NYHA functional class			
III‐IV (*n*)	19	14	0.011
6MWD (m)	438 ± 54	417 ± 63	0.033
Laboratory biomarkers			
NT‐proBNP (pg/mL)	251.9 (151.9, 867.8)	1817.7 (440.7, 2676.0)	0.003
WBC (10^9^/L)	6.8 ± 2.8	7.0 ± 2.7	0.862
Scr (μmol/L)	65.0 ± 13.1	69.1 ± 14.1	0.086
BUN (mmol/L)	5.5 ± 1.9	6.1 ± 2.7	0.114
ALT (IU/L)	14.9 (9.7, 23.0)	15.0 (9.0, 26.0)	0.349
AST (IU/L)	22.7 (18.3, 31.2)	23.9 (19.2, 31.5)	0.073
Hemodynamic measurements			
RAP (mm Hg)	7 ± 4	8 ± 5	0.050
mPAP (mm Hg)	54 ± 20	63 ± 24	0.025
CO (L/min)	4.4 ± 2.0	4.2 ± 1.5	0.466
CI (L·min^−1^·m^−2^)	2.9 ± 1.1	2.7 ± 0.9	0.339
PVRI (dyn·s·cm^−5^·m^2^)	737.2 (355.9, 1552.5)	974.4 (489.4, 1764.8)	0.117
HR (beats·min^−1^)	83 ± 14	85 ± 13	0.300
SvO_2_ (%)	68.7 ± 8.1	64.4 ± 8.8	0.003

Notes

Data are presented as numbers (percentage), median (25%‐75%) or mean ± Standard Deviation.

Abbreviations: 6MWD, 6 minutes walking distance; ALT, alanine aminotransferase; AST, aspartate aminotransferase; BMI, body mass index; BUN, blood urea nitrogen; CHD, congenital heart disease; CI, cardiac index; CO, cardiac output; CTD, connective tissue disease; HR, heart rate; HT, hypertension; mPAP, mean pulmonary artery pressure; NT‐proBNP, N‐terminal prohormone of brain natriuretic peptide; NYHA, New York Heart Association; PVRI, pulmonary vascular resistance index; RAP, right atrial pressure; Scr, serum creatinine; SvO_2_, mixed venous oxygen saturation; WBC, white blood cell; WHO, World Health Organization.

### 3.2 Univariate and multivariate cox regression

Low‐risk criteria were based on 2015 ESC/ERS guidelines: (a) WHO/NYHA functional class I‐II, (b) 6MWD > 440 m, (c) NT‐proBNP* *< 300 pg/mL, (d) RAP* *< 8 mm Hg, (e) CI ≥ 2.5 L·min^−1^·m^−2^, (f) SvO_2_ > 65%. The correlation between low‐risk criteria and AEs was analysed by Cox regression, and are reported in Table 2. In univariable model, WHO/NYHA functional class I or II, 6MWD > 440 m, NT‐proBNP* *< 300 pg/mL, and SvO_2_ > 65% were associated with prognosis. However, 6MWD > 440 m and NT‐proBNP* *< 300 pg/mL were the only remained predictors of prognosis in multivariable model.

**Table 2:** Univariable and multivariable Cox regression analysis

Low risk category	Univariable analysis			Multivariable analysis		
	HR	95% CI	*P* value	HR	95% CI	*P* value
WHO/NYHA FC I‐II	0.46	0.26‐0.86	0.015	–	–	–
6MWD > 440 m	0.06	0.03‐0.16	0.000	0.08	0.03‐0.19	0.000
RAP < 8 mm Hg	0.68	0.35‐1.29	0.233	–	–	–
CI ≥ 2.5	0.60	0.32‐1.12	0.110	–	–	–
SvO_2_ > 65%	0.38	0.21‐0.72	0.003	–	–	–
NT‐ProBNP < 300 pg/mL	0.24	0.12‐0.51	0.000	0.35	0.16‐0.78	0.010

Notes

Multivariable model was adjusted by WHO/NYHA functional class I‐II, 6MWD > 440 m, SvO_2_ > 65%, and NT‐proBNP < 300 pg/mL.

Abbreviations: 6MWD, 6 minutes walking distance; CI, cardiac index; FC, functional class; NT‐proBNP, N‐terminal prohormone of brain natriuretic peptide; RAP, right atrial pressure; WHO, World Health Organization.

### 3.3 Risk assessment

**Figure 2:**
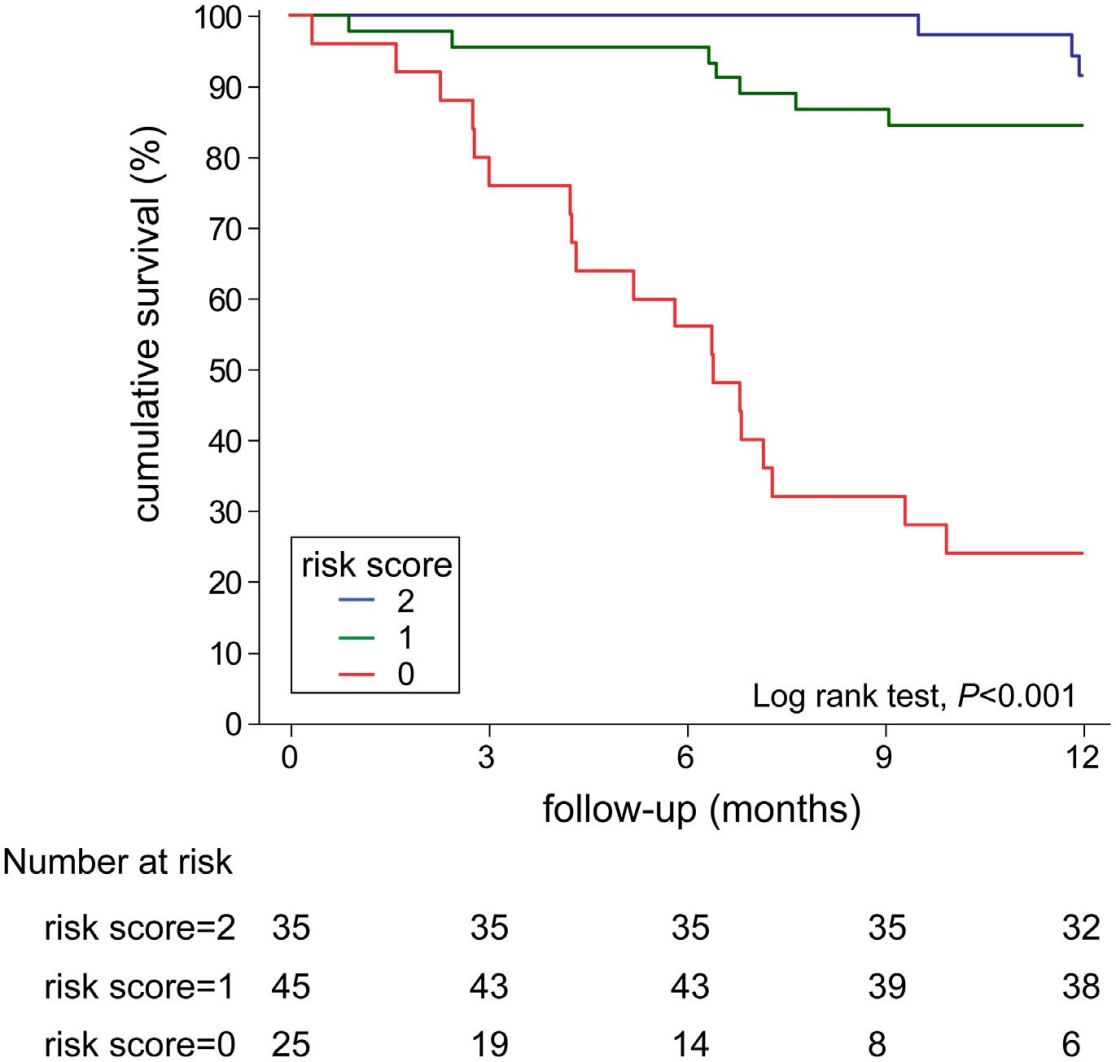
Survival by risk score. Kaplan‐Meier curves showing the cumulative survival PAH‐CHD patients grouped by risk score. Score of 0~2 was given based on the number of low‐risk criteria

**Figure 3:**
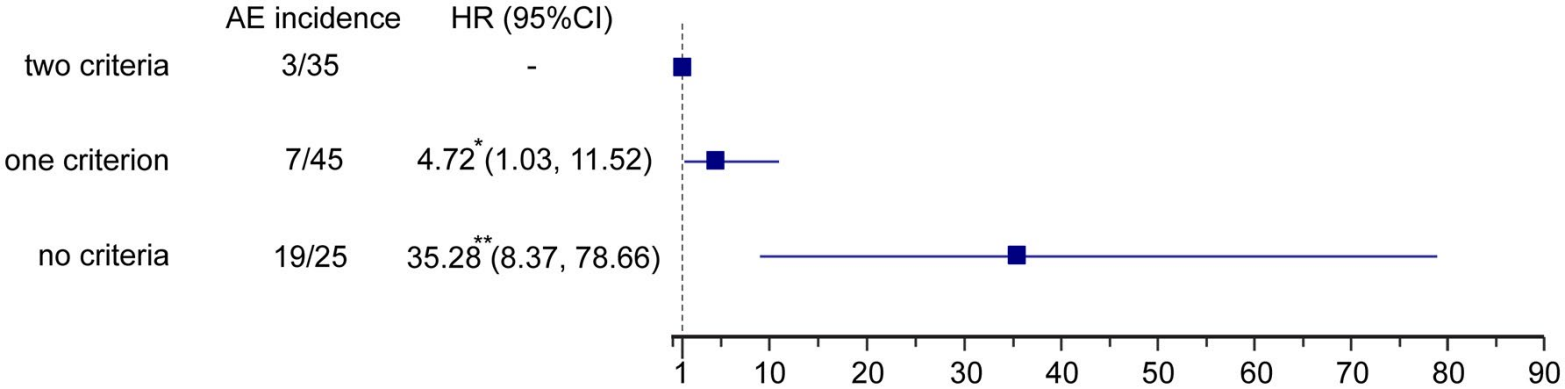
AEs risk by number of low‐risk criteria. Forest plot showing the AEs risk of PAH‐CHD patients with different number of low‐risk criteria. HR, hazard ratio; ^*^ *P *< 0.05; ^**^ *P *< 0.01

Risk assessment was performed using a simplified system that contained two independent predictors—6MWD > 440 m and NT‐proBNP* *< 300 pg/mL. A risk score was given to the patients based on their numbers of low‐risk criteria––35 (33.30%), 45 (42.90%) and 25 (23.80%) of patients had two, one or no low‐risk criteria, respectively. Figure 2 demonstrates the overall survival, according to risk score. Patients with two low‐risk criteria had the best prognosis, which included 3 (8.60%) AEs at the end of follow‐up. One criterion less meant nearly 5‐folds higher risk of AEs, whereas no low‐risk criteria at diagnosis increased risk of AEs by 35‐folds (Figure 3).

## 4 DISCUSSION

Our study was first to investigate the prognostic value of parameters proposed in 2015 ESC/ERS guidelines for Chinese patients with PAH‐CHD. Specifically, 6MWD > 440 m and NT‐proBNP* *< 300 pg/mL were the independent risk factors of poor prognosis for the study cohort. A simplified system for risk stratification containing the two independent risk factors was proposed thereafter. Patients with higher score had better prognosis. These findings suggest that (a) low‐risk criteria from guidelines need further evaluation before applying in local cohort; (b) simplified risk assessment based on guidelines provides comprehensive predictive value; and (c) PAH‐CHD patients with more low‐risk criteria at diagnosis would have better prognosis.

Current guidelines recommend a multi‐dimensional risk assessment for all PAH patients at baseline. PAH patients at high‐risk criteria have nearly 5% more mortality than those who are at low‐risk criteria.^3^ Studies also proved that multiple‐marker approach for risk assessment is better for PAH patients.^9^, ^10^ The REVEAL score first provided a comprehensive system for risk assessment in PAH patients, which included 19 parameters in 9 categories. Only 58.20% of PAH patients with very high risk were likely to survive after 12 months from enrolment.^11^ Despite of the predictive value, the REVEAL score is too complicated to apply in routine practice. Additionally, it remains uncertain whether the existed risk assessment would be effective for PAH‐CHD patients who consisted of most PAH patients in Asia.^1^

Here, we evaluated low‐risk criteria proposed by current guidelines in PAH‐CHD patients. Although WHO/NYHA functional class I or II, 6MWD > 440 m, NT‐proBNP* *< 300 pg/mL, and SvO_2_ > 65% were associated with prognosis in univariable model, only 6MWD > 440 m and NT‐proBNP* *< 300 pg/mL remained their predictive value in multivariable model. The results of our study are similar with others. Being a marker that is highly associated with right heart function, 6MWD has been widely applied in risk stratification for PAH patients regardless their subgroups.^12^, ^13^, ^14^ Though cut‐off values varied slightly among studies, the predictive value of 6MWD was consistent. NT‐proBNP is another biomarker reflecting right heart function,^15^ and it is associated with different cardiac markers such as WHO functional class, and hemodynamics.^16^, ^17^ Studies showed that NT‐proBNP was significantly associated with prognosis in either idiopathic PAH or PAH‐CHD patients.^18^, ^19^ However, single biomarker identifies patients less accurately. Therefore, studies tried to use both 6MWD and NT‐proBNP for risk stratification for iPAH, CTD‐PAH and chronic thromboembolic pulmonary hypertension (CTEPH).^20^, ^21^ It turns out that the patients at high risk of poor prognosis were identified with the two simple markers. Though with different mechanisms underling, the pathogenic progress in PAH subgroups are similar because most patients die because of the right heart failure at the end stage.^22^ The independent prediction of prognosis by 6MWD and NT‐proBNP in our study expanded the value of these two parameters in risk assessment for PAH‐CHD, and further strengthened the critical role of right heart function for PAH patients regardless their subgroups. The predictive value of some other markers, such as functional class, RAP and CI, was eliminated in our study, which might be the results of being adjusted by 6MWD and NT‐proBNP and different etiology.

In present study, a simplified system of risk stratification based on current guidelines is successfully identified PAH‐CHD patients with poor prognosis. With only two criteria, 6MWD and NT‐proBNP, both of which predicted prognosis independently, patients were separated into three groups by numbers of low‐risk criteria with different risk of AEs. Patients had two low‐risk criteria at baseline archived 97% AE‐free survival during one‐year follow‐up. With one low‐risk criteria, AE‐free survival reduced to 88% but no significantly different. No low‐risk criteria at diagnosis increased AE risk by 35 folds and only 37% patients were AE‐free during follow‐up. While applying, only one criteria above identified patients at risk successfully but with lower sensitivity.^9^ Risk assessment for PAH patients is a continuous process, which should be performed at the time of diagnosis and during follow‐up.^23^ However, accurate risk stratification prior to the treatment first identifies the patients with poor prognosis and guides medical care thereafter. Therefore, our study following previous study shows that simplified risk stratification at diagnosis according to current guidelines, even with only two criteria, could accurately identifies PAH‐CHD patients at risk during one‐year follow‐up.^24^ Notably, two criteria in our study were both noninvasive parameters. Although hemodynamic parameters are important for PAH‐CHD diagnosis and re‐evaluation during follow‐up,^25^ the accurate predictive of prognosis by noninvasive parameters might offer a better solution for risk stratification when right catheterisation is not available.

### 4.1 Limitation

There are several limitations in our study. First of all, this is a single centre study. Incomplete information leads to a relatively small study population, and small number in each subgroup, which may reduce the statistic power of RAP, CI and others. Second, our retrospective observational study doesn’t offer a long‐term follow‐up. Few AEs during short‐term follow‐up may further eliminate significance of some predictors. Further study may expand the study population and follow‐up to provide more evidence. Third, only patients older than 18 were included in our study. However, risk assessment is also critical for young patients with PAH‐CHD (<18 years). We may focus on those who are younger than 18 in future investigation.

In conclusion, low‐risk criteria of 6MWD > 400 m and NT‐proBNP* *< 300 pg/mL according to 2015 ESC/ERS guidelines were significantly associated with less AEs in PAH‐CHD patients during one‐year follow‐up. In addition, simplified risk assessment will successfully identify PAH‐CHD patients with poor prognosis at admission.

## CONFLICT OF INTEREST

The authors declared that they have no conflicts of interest with the contents of this article.

## AUTHOR CONTRIBUTIONS

*Designed the study:* Zheng and Zhang

*Collected clinical data:* Deng, Jin, Li, Li and Zhou

*Laboratory tests and analysis:* Wu, Hu and Qiu

*Statistical analysis:* Yan

*Study and wrote the paper:* Deng and Zheng

## ETHICS

The study was approved by the Ethics Committee of Wuhan Asia heart hospital with a waiver of informed consent (2014‐P‐001). Personal information of patients was re‐identified before analysis.
